# Direct estimates of national neonatal and child cause–specific mortality proportions in Niger by expert algorithm and physician–coded analysis of verbal autopsy interviews

**DOI:** 10.7189/jogh.05.010415

**Published:** 2015-06

**Authors:** Henry D. Kalter, Abdoulaye–Mamadou Roubanatou, Alain Koffi, Robert E. Black

**Affiliations:** 1 Department of International Health, Johns Hopkins Bloomberg School of Public Health, Baltimore, MD, USA; 2 Ministry of Health, Niamey, Niger; 3 The Institute for International Programs, Johns Hopkins Bloomberg School of Public Health, Baltimore, MD, USA

## Abstract

**Background:**

This study was one of a set of verbal autopsy investigations undertaken by the WHO/UNCEF–supported Child Health Epidemiology Reference Group (CHERG) to derive direct estimates of the causes of neonatal and child deaths in high priority countries of sub–Saharan Africa. The objective of the study was to determine the cause distributions of neonatal (0–27 days) and child (1–59 months) mortality in Niger.

**Methods:**

Verbal autopsy interviews were conducted of random samples of 453 neonatal deaths and 620 child deaths from 2007 to 2010 identified by the 2011 Niger National Mortality Survey. The cause of each death was assigned using two methods: computerized expert algorithms arranged in a hierarchy and physician completion of a death certificate for each child. The findings of the two methods were compared to each other, and plausibility checks were conducted to assess which is the preferred method. Comparison of some direct measures from this study with CHERG modeled cause of death estimates are discussed.

**Findings:**

The cause distributions of neonatal deaths as determined by expert algorithms and the physician were similar, with the same top three causes by both methods and all but two other causes within one rank of each other. Although child causes of death differed more, the reasons often could be discerned by analyzing algorithmic criteria alongside the physician’s application of required minimal diagnostic criteria. Including all algorithmic (primary and co–morbid) and physician (direct, underlying and contributing) diagnoses in the comparison minimized the differences, with kappa coefficients greater than 0.40 for five of 11 neonatal diagnoses and nine of 13 child diagnoses. By algorithmic diagnosis, early onset neonatal infection was significantly associated (χ^2^ = 13.2, *P* < 0.001) with maternal infection, and the geographic distribution of child meningitis deaths closely corresponded with that for meningitis surveillance cases and deaths.

**Conclusions:**

Verbal autopsy conducted in the context of a national mortality survey can provide useful estimates of the cause distributions of neonatal and child deaths. While the current study found reasonable agreement between the expert algorithm and physician analyses, it also demonstrated greater plausibility for two algorithmic diagnoses and validation work is needed to ascertain the findings. Direct, large–scale measurement of causes of death complement, can strengthen, and in some settings may be preferred over modeled estimates.

Health policy makers and program planners require data on the levels and causes of death in order to identify health priorities, allocate sparse resources and evaluate health program impact. Death registration with medical certification of the cause of death is the best source of such data, but a minority of low and middle income counties (LMIC) have well–functioning vital registration systems with good population coverage, and in many countries a large proportion of deaths occur outside of medical care. In such settings, verbal autopsy interviews conducted in Demographic and Surveillance Sites [1], as part of a national survey [2], or in a few countries in nationally–representative sample registration systems [3], remains the best source of empirical data on causes of death.

A verbal autopsy (VA) inquiry of a child death consists of a retrospective interview on the signs and symptoms of the fatal illness with the mother or other main caregiver of the child. The cause of death is determined from pre–defined, expert–determined, combinations of the reported illness signs and symptoms (algorithms) or by independent classification of the VA interview findings by one or more physicians. The method has been directly validated against medical reference standard diagnoses and has been found to work best in identifying distinctive syndromes such as tetanus, measles and injuries and moderately well for less specific illnesses like pneumonia and malaria [4-9]. Newer, statistical and probabilistic analytic approaches have shown promise in increasing the validity of verbal autopsy diagnoses [10], but up till now these methods have not been directly compared to VA algorithms and agreement has not been reached on the best analysis method [11]. Also, widely accepted and user–friendly software needed to conduct statistical analyses of VA data has yet to be produced and made accessible.

As part of the Child Health Epidemiology Reference Group’s (CHERG) recent effort to directly measure the causes and determinants of neonatal and child mortality in selected, high–priority countries, a national verbal/social autopsy (VASA) study was conducted in Niger. Niger was selected because its child mortality level is among the highest in the world, ranked number 10 in under–5 mortality [12]; because a recent national mortality survey demonstrated that there has been a significant decrease in under–5, but not neonatal, mortality; and because there were no previous reliable or large–scale direct measures of the causes of neonatal or child deaths in Niger. In addition to the concern of global public health practitioners, both the Ministry of Health of Niger and the UNICEF country office took a keen interest in the study and have utilized the findings in the development of improved maternal and child health policies and programs. This paper reports on the verbal autopsy findings of the VASA study.

## METHODS

### Study sample

The deaths included in the Niger VASA study were identified by the Niger National Mortality Survey (NNMS) conducted in July to August 2010. This survey used a two–stage random cluster design to select 25 024 households. A lifetime birth history was conducted for all women 15 to 49 years old in each sampled household to identify all live births and child deaths [13,14].

The VASA study sought to examine samples of the most recent 605 neonatal (0 to 27 days old) and 605 child (1 to 59–month olds) deaths, which, with alpha = 0.05, Z = 1.96, design effect = 1.4 and non–response rate = 0.1, are sufficient to achieve precision of ±0.05 around an assumed proportion of 0.50 for the most common cause of death in each age group. This required sampling deaths as far back from the survey period as four years, during which there were 734 neonatal deaths and 1646 child deaths. From these, starting with the most recent under–five years old death (whether it was a neonate or child) in all the households and moving back in time, we selected the one most recent under–five years old death (or one at random if there were two or more most recent deaths in the same month) in each household with at least one such death until we had achieved our desired sample sizes of 605 deaths in each age group. Comparing this method with selecting one death at random from each household in the same time period showed no substantial differences in the child’s age at death or sex or in the respondent’s age. We therefore took the most recent deaths in order to limit the recall period as much as possible, while maintaining the representativeness for each age group within the time period covered by the deaths in that group.

### VASA interview

The VASA questionnaire developed for this study blends the Population Health Metrics Research Consortium (PHMRC) verbal autopsy questionnaire [15] with the CHERG social autopsy questionnaire [16]. The original English VASA questionnaire was translated to French and then from French to the two main languages of Niger, Haoussa and Zarma. Each Nigerien language questionnaire was independently back–translated to French to cross–check and reconcile the translations, and then scrutinized by a local anthropologist to ensure that appropriate local terms were used for the illness signs and symptoms.

The translated questionnaires were inserted into a CSProX [17] CAPI (computer–assisted personal interview) software application developed for the VASA studies, and the interviews were conducted and responses captured in the field directly on netbook computers. The software was designed to minimize data entry errors by guiding the interviewer through the questionnaire and providing numerous real–time data checks and opportunities to correct internally inconsistent responses.

The VASA interviews were conducted as follow–up visits to the households with a death identified by the NNMS. Most of the fieldwork was conducted from March to April 2012. Revisits to 114 households to resolve discrepancies in the deceased children's birth and death dates determined by the VASA and NNMS extended the data collection until September 2012.

The interviewers were 12 women and eight men, all native speakers of Haoussa and/or Zarma, 86% of whom had some post–secondary education and the remaining had completed secondary school. They received 10 days of classroom training in the VASA study background, procedures, ethical standards and conduct of the interview on the netbook, followed by three days of field practice, all conducted in French, Haoussa and Zarma. Each of the seven teams of two to four interviewers and one supervisor was visited twice by an office supervisor during the 55 days of data collection to provide additional supervision and to collect interim copies of the data files for monitoring purposes.

The interviewers were trained to select as the respondent the person who most closely cared for the child during the fatal illness, which is typically, but not always, the child’s mother. Secondary respondent(s) were allowed, if necessary, since the interview covered all phases of the illness and careseeking including, for neonatal deaths, the mother’s pregnancy and delivery, during and after which she herself might have been ill and so less aware of the child’s condition and illness events. In case of any disagreement between respondents, the main respondent’s answer was always taken as final.

### Development of verbal autopsy algorithms and hierarchies

The expert algorithms (EAVA) for neonatal and child causes of death used by this study (see **Online Supplementary Document(Online Supplementary Document)**) utilized questions in the VA portions of the VASA questionnaire and one social autopsy question on the ordering of onset of the illness signs and symptoms. The algorithms were based on those developed by verbal autopsy researchers for prior VA validation studies [5-9], further consultation with additional verbal autopsy experts (GD and AB in acknowledgments), and a literature review to identify illness signs and symptoms commonly associated with particular neonatal and child illnesses [18-21]. Algorithms for some conditions included in this study have not been developed or tested in prior validation studies; new algorithms were developed for these conditions, including neonatal jaundice, neonatal hemorrhagic syndrome, AIDS and hemorrhagic fever. The Pertussis algorithm was adapted from the US Centers for Disease Control and Prevention case definition [22].

Most algorithms were selected for their expected higher specificity than sensitivity in order to decrease false positives, as this characteristic minimizes misclassification error in the VA diagnosis of neonatal and child causes of death in developing countries [23]. Algorithms for possible pneumonia or acute respiratory infection (ARI), possible diarrhea, possible dysentery and possible malaria, all designed to have higher sensitivity than their corresponding probable diagnoses, were developed to claim these possible diagnoses from the unspecified cause of death group. The final cause of death distributions combined probable and their related, possible, diagnoses.

In addition to algorithms for neonatal and child causes of death, an algorithm for one maternal condition, infection before or during labor and delivery (see **Online Supplementary Document(Online Supplementary Document)**), was developed to assess the association between maternal infection and early onset severe neonatal infection.

Hierarchies were developed for the neonatal and child diagnoses (see **Online Supplementary Document(Online Supplementary Document)**) to select the EAVA primary cause of death for each child; the hierarchies also allowed for the identification of possible co–morbid causes. In addition, if a child had a diagnosis either as the primary or a co–morbid cause of death, then that was assigned as an overall ‘algorithmic cause’, which was compared to the overall physician–certified VA diagnoses as described below.

The ordering of the hierarchies was based mainly on principles incorporated in the ICD–10 rules of identifying the main disease or condition of the infant for early neonatal deaths (referred to in the ICD rules, together with stillbirths, as perinatal deaths, and recorded on a separate perinatal certificate), and for older infants and children the underlying cause of death, meaning the condition as a consequence of which the direct cause of death occurred [24]; and for some conditions to select the most severe or site–specific morbidity as the primary cause. The ICD–10 rules for perinatal deaths specify that the mode of death, including prematurity, should not be classified as the main disease or condition of the infant unless it was the only condition known. This rule was followed by placing preterm delivery at the bottom of the hierarchy for neonatal deaths, in order to select possible co–morbid conditions such as sepsis as the main disease or condition. An example of the underlying cause principle is that in the child hierarchy measles was placed above pneumonia because pneumonia is likely to have occurred as a consequence of measles in a child with both conditions; and of the severity or site–specific principle is that in the neonatal hierarchy meningitis was placed above sepsis because it identifies the focus of the infection.

This last example above also illustrates how the hierarchies identify possible comorbidity as well as the main or underlying cause of death. A neonate with meningitis would most likely also have sepsis; in such a case the hierarchy would first select meningitis, placed above sepsis, as the primary cause, and below would identify co–morbid sepsis. Diarrhea was placed above pneumonia in the hierarchies based on WHO’s interpretation of the ICD–10 rule that pneumonia should be considered a consequence of conditions that impair the immune system [25]. Lastly, all possible diagnoses were placed below their corresponding probable diagnoses to detect possible cases that did not meet the probable cause criteria.

The hierarchies developed for this study differ somewhat from the standardized CHERG hierarchy developed for an earlier study that examined trends in the causes of child mortality in Bangladesh [26]. In addition to incorporating several conditions not included in the earlier hierarchy (for neonates: meningitis, neonatal jaundice, hemorrhagic disease of the newborn and sudden unexplained death; for 1 to 59–month olds: AIDS, dysentery, Pertussis, malaria and hemorrhagic fever), the current neonatal hierarchy moved preterm delivery below all other conditions in accordance with the ICD rule cited above, the child hierarchy moved malnutrition up in order to identify malnutrition as an underlying cause of death, and both the neonatal and child hierarchies identified diarrhea ahead of pneumonia, in keeping with WHO’s interpretation of the ICD rule for coding pneumonia in the presence of conditions that impair immunity.

### Physician cause of death assignment

One physician, a Nigerien neonatologist (A–MR), read the VA interviews and completed an international certificate of death for each neonatal and child death. Guidelines for classifying the cause of death from a VA interview, including minimal diagnostic criteria required for each cause (see **Online Supplementary Document(Online Supplementary Document)**), were developed for the physician’s use together with her clinical judgment and discussed in a three–hour training session. WHO standards for attributing cause of death from verbal autopsy [25] also were discussed during the training, and the physician was provided a copy of both documents. The physician completed several practice cases prior to starting the work, which were reviewed and discussed with her to help ensure proper filling of the death certificates.

The underlying cause of death, which is the antecedent cause on the lowest of lines 1a to 1d of section 1 of the filled certificate, was taken as the physician–certified (PCVA) cause of death. Any other causes listed higher in the causal chain, as well as any contributing causes of death listed in section 2 of the certificate, also were recorded. For neonates, the physician also certified any maternal underlying and contributing causes of the neonatal death. Any maternal underlying causes (in section 1 of the death certificate) always were placed beneath the child cause(s). In such cases, the child cause lowest in lines 1a to 1c was taken as the underlying cause of death, and the maternal cause lowest in lines 1b to 1d was the underlying maternal cause. An example would be a neonatal death with birth asphyxia as the underlying cause of death in line 1a and obstructed labor as the underlying maternal cause in line 1b. Although a separate perinatal certificate was not utilized to classify neonatal deaths, the examples provided in the physician’s guide and in the WHO VA standards manual make clear that preterm delivery should not be coded as the underlying cause of death when another condition is present.

All direct, antecedent and contributing child causes for each cause of death were combined into one overall ‘physician cause’ if the child had that diagnosis at any of the three levels, which was compared to the combined primary and co–morbid ‘algorithmic cause’ in order to assess the overall level of agreement between the algorithmic and physician diagnoses of the child causes of death.

### Meningitis surveillance data

Surveillance data on all–ages meningitis cases and deaths in 2007 to 2010, stratified by the country’s eight regions, were available from the Niger Ministry of Health’s Centre for Medical Research and Health, which works closely with the Institute Pasteur. These data were used to conduct an ecological plausibility check of the VA diagnoses of child meningitis deaths. The surveillance data for 2007 to 2009, during which 86% of the total 21 898 cases occurred, included the number of cases notified, the number for which the public health laboratory received a cerebrospinal fluid (CSF) sample, and the number of samples with a positive bacterial culture. The 2010 data included only the number of cases notified.

### Statistical analyses

The EAVA diagnostic criteria and hierarchies were computerized to automate the determination of the distributions of neonatal and child primary causes of death and possible co–morbid causes from the VA interview responses. The PCVA diagnoses of direct, underlying and contributing causes of death were directly entered into the computer.

The rank ordering of EAVA primary causes of death and PCVA underlying causes was separately compared for the neonatal and child deaths. Differences between the mortality proportions for each EAVA primary cause of death and PCVA underlying cause of death were evaluated with the mid–p chi–square test of proportions [27] and by examining the overlap of their 95% confidence intervals. The level of agreement beyond that due to chance alone between the combined ‘algorithmic cause’ and the overall ‘physician cause’ of death diagnoses was assessed with the Kappa statistic [28] in order to evaluate the degree to which differences in the primary EAVA and PCVA diagnoses were due to the ordering of the diagnoses by the EAVA hierarchy and the physician. The chi–square statistic was used to evaluate the association between maternal infection and early onset severe neonatal infection, including meningitis, pneumonia and sepsis separately and combined. All statistical analyses were performed using SAS version 9.2 for Windows [29]. Because the deaths analyzed in this study were identified by the NNMS, the survey sample cluster weights were applied to all analyses, including determination of the EAVA and PCVA cause–specific mortality proportions, the level of agreement between the EAVA and PCVA diagnoses, and the associations between maternal infection and early onset neonatal infection.

The relationship between the geographic distributions of EAVA and PCVA child meningitis deaths and all–ages meningitis surveillance cases and deaths was examined by comparing the VA–diagnosed region–meningitis–specific proportional mortality for 1 to 59–month old children to the percentage of the entire country’s surveillance–detected all–ages meningitis cases and deaths in each region. We examined the child VA meningitis–specific proportional mortality in each region instead of the percent of all child VA meningitis deaths that occurred in each region because, just as for the surveillance data, this could be influenced by the regional population distribution (**Table 1**), ie, given similar attack rates, the more people in a region, the more cases and deaths from any particular cause might be expected. This assessment examined only the primary EAVA and underlying PCVA cause of death since the purpose was to evaluate the meningitis diagnoses that could be reported for vital statistics purposes.

**Table 1:** Distribution of the verbal–social autopsy deaths (VASA), by region of Niger

		VASA cases		
**Region**	**2007–2010 population**	**Neonatal deaths**	**Child deaths**	**Total**
Agadez	420 026	21	8	29
Diffa	472 799	43	43	86
Dosso	1 944 322	81	116	197
Maradi	2 942 972	79	94	173
Tahoua	2 536 713	51	104	155
Tillabéri	2 416 875	84	95	179
Zinder	2 683 738	76	151	227
Niamey	954 613	18	9	27
Total	14 372 058	453	620	1073

### Ethics approval

The study was approved by the National Consultative Ethics Committee of the Niger Ministry of Health and by the Institutional Review Board of the Johns Hopkins Bloomberg School of Public Health. All the study personnel received training in ethical principles and practices for human subjects research, and informed consent was given by all study participants before the VASA interview was conducted.

## RESULTS

The final VASA sample consisted of 1166 (96.9%) completed interviews of 1203 attempted, including 453 neonatal deaths, 620 child deaths and 93 stillbirths. The 93 stillbirths derived from the VASA interviews determining that these (primarily) neonatal deaths of live born children identified by the NNMS were in fact stillbirths. Because the NNMS was not designed to detect stillbirths, and so these deaths do not constitute a representative sample of stillbirths, they were not included in the current analysis. In addition to the live births identified by the NNMS that were determined by the VASA to be stillbirths, some additional cases moved between the neonatal and child age groups. These were double–checked during revisits to the affected households. The final VASA–determined birth status and age at death were taken as the correct data for this study. **Table 1** shows the geographic distribution of the neonatal and child deaths.

The interview recall periods (from death till the VASA interview) for the neonatal and child deaths were, respectively, 2 to 5 years (mean = 3.51, standard deviation = 1.06 years) and 2 to 5 years (mean = 2.69, standard deviation = 0.88 years). Three–hundred eleven (68.7%) of the 453 neonates died before attaining seven days of age. Of the 620 children, 269 (43.4%) died at age 1 to 11 months, 144 (23.2%) at 12 to 23 months old, and 207 (33.4%) at age 24 to 59 months.

### Neonatal deaths

**Causes of death.** **Table 2** shows the EAVA primary and possible co–morbid causes of death of the 453 neonates. Taking sepsis, pneumonia and meningitis together, the primary cause of death of 240 (53.2%) of the neonates was a severe infection. Another 18 (4.0%) died from tetanus and 26 (5.7%) from diarrhea. After infectious causes, the next leading primary condition was birth injury and/or asphyxia, causing 90 (19.9%) of the deaths. Preterm delivery was the primary cause of death of only 12 (2.7%) of the neonates; and including all cases with either primary or co–morbid preterm, only 41 (9.1%) of the newborns had preterm delivery as a cause of death. The EAVA analysis was not able to classify the cause of death for 42 (9.2%) of the neonates.

**Table 2:** Expert algorithm, hierarchical verbal autopsy primary and possible co–morbid causes of 453 neonatal deaths, Niger, 2007–2010

EAVA primary cause of death (possible co–morbid causes)	N	%
Neonatal tetanus (5 BI/BA, 3 diarrhea, 3 pneumonia)	18	4.0
Congenital malformation (8 BI/BA, 1 meningitis, 1 diarrhea, 3 pneumonia, 2 preterm, 9 sepsis)	12	2.7
Birth injury (9) and/or asphyxia (85) (6 meningitis, 1 diarrhea, 34 pneumonia, 9 preterm, 78 sepsis, 2 hemorrhagic disease)	90	19.9
Meningitis (14 pneumonia, 19 sepsis)	19	4.2
Diarrhea (4 pneumonia, 26 sepsis)	26	5.7
Pneumonia (53 sepsis)	53	11.6
Sepsis (17 preterm)	169	37.4
Neonatal jaundice	1	0.2
Hemorrhagic disease of the newborn	1	0.3
Sudden unexplained death (1 preterm)	10	2.1
Preterm delivery (7 with Respiratory Distress Syndrome)	12	2.7
Unspecified	42	9.2

EAVA – expert algorithm verbal autopsy, BI/BA – birth injury and/or birth asphyxia

**Table 2** also demonstrates a high degree of possible comorbidity, with sepsis being the most common co–morbid condition, particularly found in most deaths caused by birth asphyxia and in all cases of meningitis, pneumonia and diarrhea. Preterm delivery was another common co–morbid condition, occurring in 17 cases of primary sepsis and 9 of birth asphyxia.

**Figure 1:**
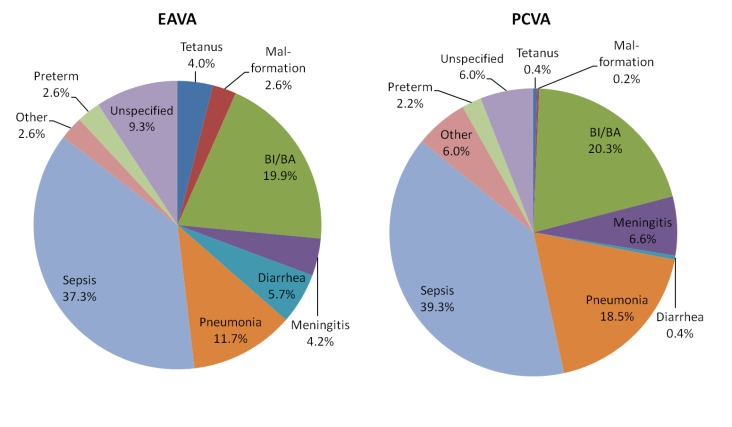
Verbal autopsy expert algorithm, hierarchical primary and physician–certified underlying causes of 453 neonatal deaths, Niger, 2007–2010. EAVA – expert algorithm verbal autopsy, PCVA – physician–certified verbal autopsy. Other: EAVA – 1 neonatal jaundice, 1 hemorrhagic disease of the newborn, 10 sudden unexplained death; PCVA – 3 neonatal jaundice, 12 sudden unexplained death, 12 refuse to suck.

**Figure 1** shows the neonates’ EAVA primary causes of death and PCVA underlying causes of death. Leaving aside deaths with an unspecified diagnosis, both methods ranked sepsis, birth injury/asphyxia and pneumonia, respectively, as causes 1, 2 and 3, and all but two other causes (diarrhea and other) were within one rank of each other. While the relative proportions of the causes and the overall pictures are quite similar, **Table 3** reveals some substantial differences in the proportion of deaths due to several individual causes. Severe neonatal infections predominated by both analytic methods, with each identifying more sepsis than pneumonia and more pneumonia than meningitis. The largest difference was in the higher overall percentage of severe infections diagnosed by PCVA (64.4%) compared to EAVA (53.2%), and the correspondingly lower percentage of PCVA diarrhea and tetanus deaths (combined, 0.8% vs 9.7% for the algorithms). Also, PCVA failed to classify the cause of 6.0% of the deaths, compared to 9.2% for EAVA.

**Table 3:** Expert algorithm– and physician–diagnosed primary cause of death mortality proportions, 95% confidence limits, chi–squares and p–values for 453 neonatal deaths, Niger, 2007–2010

Diagnosis	EAVA (%)	95% CL*	PCVA (%)	95% CL*	χ^2^	P
Neonatal tetanus	4.0	2.4, 6.1	0.4	0.1, 1.5	13.1	<0.001
Malformation	2.6	1.4, 4.5	0.2	0.01, 1.1	9.4	0.002
Birth injury and/or asphyxia	19.9	16.4, 23.7	20.3	16.8, 24.2	0.03	0.860
Meningitis	4.2	2.6, 6.4	6.6	4.6, 9.2	2.6	0.106
Diarrhea	5.7	3.9, 8.2	0.4	0.1, 1.5	21.2	<0.001
Pneumonia	11.5	8.8, 14.7	18.5	15.2, 22.3	8.9	0.003
Sepsis	37.3	32.9, 41.8	39.3	34.9, 43.9	0.4	0.538
Sepsis + Pneumonia + Meningitis	53.0	48.4, 57.6	64.4	60.0, 68.8	12.3	<0.001
Other†	2.6	1.4, 4.5	6.0	4.0, 8.4	6.0	0.014
Preterm	2.7	1.4, 4.5	2.2	1.1, 3.9	0.2	0.666
Unspecified	9.3	6.9, 12.2	6.0	4.0, 8.4	3.5	0.060

EAVA – expert algorithm verbal autopsy, PCVA – physician–certified verbal autopsy

*Mid–p 95% confidence limits.

†Other: neonatal jaundice, hemorrhagic disease of the newborn and sudden unexplained death.

**Table 4** shows the level of agreement between the combined ‘algorithmic cause’ and overall ‘physician–cause’ of death for each diagnosis. There was excellent agreement, with a kappa greater than 0.80, for two of the 11 causes, good agreement, with a kappa greater than 0.60, for two causes, moderate agreement, with a kappa greater than 0.40, for one cause, and fair agreement, with a kappa greater than 0.20, for three causes. Only two causes (tetanus and jaundice) had less than chance agreement, with the lower 95% confidence limit below 0.

**Table 4:** Agreement of all combined expert algorithm verbal autopsy (VA) primary and possible co–morbid diagnoses with all combined physician–certified VA direct, underlying and contributing causes of 453 neonatal deaths, Niger, 2007–2010

Diagnosis EAVA / PCVA	EAVA	PCVA	Agree +	Agree –	Kappa†	95%CL*
Tetanus / Tetanus	18	8	2	429	0.10	–0.07, 0.28
Malformation / Malformation	12	11	7	436	0.56	0.31, 0.80
Birth injury and/or asphyxia / BI/BA*	103	113	84	321	0.71	0.63, 0.78
Meningitis / Meningitis	26	34	9	402	0.24	0.09, 0.40
Diarrhea / Diarrhea	31	24	20	418	0.71	0.57, 0.85
Pneumonia / Pneumonia	110	87	48	305	0.35	0.25, 0.45
Sepsis / Sepsis	354	185	171	86	0.22	0.15, 0.28
Jaundice / Jaundice	3	11	2	441	0.28	–0.03, 0.59
Sudden death / Sudden death	10	12	10	441	0.88	0.74, 10.0
Preterm / Preterm	41	32	31	410	0.82	0.72, 0.92
Preterm / Refuse to suck	41	19	7	400	0.20	0.05, 0.35
Unspecified / Unspecified	42	28	13	396	0.32	0.17, 0.47

EAVA – expert algorithm verbal autopsy, PCVA – physician–certified verbal autopsy, Bi/BA – birth injury and/or asphyxia

*Mid–p 95% confidence limits.

†Kappa agreement: Less than chance ≤0, Slight ≤0.20, Fair ≥0.21, Moderate ≥0.41, Good ≥0.61, Excellent ≥0.81.

**Association of neonatal severe infection and maternal sepsis.** **Table 5** demonstrates a positive association between early onset severe neonatal infection as the primary cause of neonatal death and maternal infection, both diagnosed by EAVA. The strongest relationship between neonatal and maternal infection was for all (meningitis, pneumonia and sepsis) early onset severe neonatal infections combined compared to all later onset severe neonatal infections (χ^2^ = 13.20, *P* = 0.0003); and the weakest was for early onset pneumonia compared to later onset pneumonia. The association between early onset neonatal infection and maternal infection also was significant when comparing early onset infections to all other causes of neonatal death (χ^2^ = 6.45, *P* = 0.011).

**Table 5:** Relationship of maternal sepsis during pregnancy or delivery and early onset severe neonatal infection as the primary cause of death for 453 neonatal deaths in Niger, 2007–2010

EAVA cause of death (maternal sepsis, MS)	N	%	χ^2^, P
**Meningitis** (6 with maternal sepsis)	19	4.2	
Illness onset <2 d (MS: 5 [60.3%])	8	1.8	5.67, 0.017
Illness onset ≥2 d (MS: 1 [9.3%])	11	2.4	
**Pneumonia** (14 with maternal sepsis)	53	11.6	
Illness onset <2days (MS: 7 [30.3%])	23	5.1	0.15, 0.701
Illness onset ≥2 d (MS: 8 [25.6%])	30	6.6	
**Sepsis** (25 with maternal sepsis)	169	37.4	
Illness onset <2 d (MS: 17 [26.1%])	65	14.2	11.08, 0.001
Illness onset ≥2 d (MS: 8 [7.5%])	105	23.2	
**Severe neonatal infection** (MS: 45 [18.7%])	241	53.2	
Illness onset <2 d (MS: 28 [30.0%])	95	21.0	13.20, <0.001
Illness onset ≥2 d (MS: 17 [11.3%])	146	32.2	
**All neonatal deaths** (MS: 93 [20.6%])	453	100.0	
Severe infection onset <2 d (MS: 28 [30.0%])	95	21.0	6.45, 0.011
All other deaths (MS: 65 [18.1%])	358	79.1	

EAVA – expert algorithm verbal autopsy

### Child deaths

**Causes of death.** **Table 6** shows the EAVA primary and possible co–morbid causes of death of the 620 children aged 1 to 59 months. Malaria was the leading cause, followed by diarrhea and meningitis. Pneumonia placed a distant fourth, followed by dysentery and AIDS. Together, these six major infectious causes were responsible for 543 (87.6%) of the child deaths. Injuries caused only 6 (0.9%) deaths, and unspecified causes accounted for 28 (4.5%) deaths. Malnutrition was the underlying cause of only 14 (2.3%) deaths, but including its role as a co–morbid condition, malnutrition contributed to 145 (23.4%) of the deaths.

**Table 6:** Expert algorithm, hierarchical verbal autopsy primary and possible co–morbid causes of 620 child deaths, Niger, 2007–2010

EAVA primary cause of death (possible co–morbid causes)	N	%
Injury (2 malaria)	6	0.9
AIDS (12 meningitis, 7 dysentery, 9 diarrhea, 1 pertussis, 17 pneumonia, 3 malaria)	17	2.8
Malnutrition (1 meningitis, 3 malaria, 3 diarrhea, 2 pneumonia)*	14	2.3
Measles (3 meningitis, 2 dysentery, 4 diarrhea, 4 pneumonia, 2 malaria)	9	1.4
Meningitis (18 dysentery, 56 diarrhea, 8 pertussis, 56 pneumonia)	113	18.3
Dysentery (10 pneumonia, 7 malaria)	39	6.3
Diarrhea (40 pneumonia, 11 malaria)	121	19.5
Pertussis (2 pneumonia, 2 malaria)	2	0.3
Pneumonia (3 dysentery, 6 diarrhea, 14 malaria)	73	11.8
Malaria (8 dysentery, 20 diarrhea, 40 pneumonia)	180	28.9
Hemorrhagic fever	5	0.9
Other infections	13	2.1
Unspecified	28	4.5

EAVA – expert algorithm verbal autopsy

*131 additional cases with co–morbid malnutrition.

As with the neonates, **Table 6** also demonstrates a high degree of possible comorbidity. However, while sepsis was the predominant co–morbid condition for neonates, in children co–morbidity of pneumonia and diarrhea with primary meningitis, and between malaria, diarrhea and pneumonia were most important.

**Figure 2:**
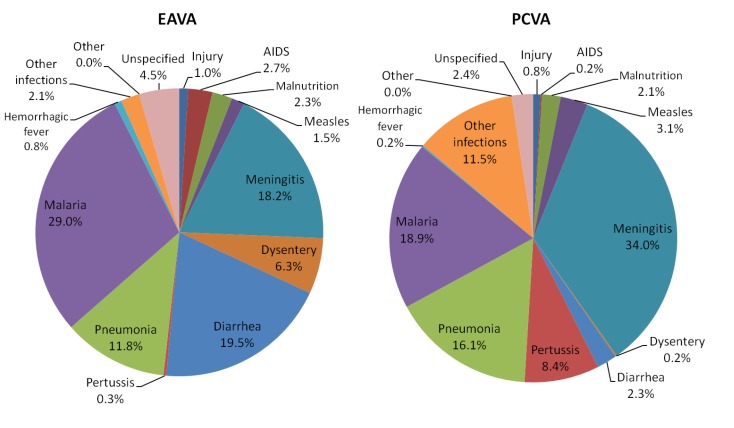
Verbal autopsy expert algorithm, hierarchical primary and physician–certified underlying causes of 620 child deaths, Niger, 2007–2010. EAVA – expert algorithm verbal autopsy, PCVA – physician–certified verbal autopsy.

**Figure 2** shows the children’s EAVA primary causes of death and PCVA underlying causes of death. Unlike for the neonates, the ranks of no leading EAVA and PCVA causes exactly matched each other, although two, malaria and pneumonia, came within one rank of each other and meningitis was ranked number 3 by EAVA and 1 by PCVA. At the low end, EAVA and PCVA both ranked injury, hemorrhagic fever and other, respectively, at number 9, 10 and 12. It was in the middle ground that the two methods most disagreed with each other, with combined diarrhea/dysentery off by five ranks, the order of measles and malnutrition reversed at ranks 6 and 8, and other infections and AIDS off, respectively by three and five ranks. Several apparent marked differences in the EAVA and PCVA proportions were confirmed by the statistical measures in **Table 7**. Malaria and pneumonia together caused, respectively, 40.8% and 35.0% of the EAVA and PCVA deaths, while the EAVA and PCVA malaria proportions alone differed markedly. There were large differences as well for several other diagnoses, with the most notable disparities being that EAVA identified more diarrhea (19.5% vs 2.3% for PCVA) and dysentery (6.3% vs 0.2%), while PCVA diagnosed more meningitis (34.0% vs 18.2% for EAVA), other infections (11.5% vs 2.1%) and Pertussis (8.4% vs 0.3%).

**Table 7:** Expert algorithm– and physician–diagnosed primary cause of death mortality proportions, 95% confidence limits, chi–squares and p–values for 620 child deaths, Niger, 2007–2010

Diagnosis	EAVA (%)	95%CL*	PCVA (%)	95% CL*	χ^2^	P
Injury	1.0	0.4, 2.0	0.8	0.3, 1.8	0.10	0.762
AIDS	2.7	1.7, 4.3	0.2	0.0, 0.8	14.4	<0.001
Malnutrition	2.3	1.3, 3.7	2.1	1.2, 3.5	0.04	0.846
Measles	1.5	0.7, 2.6	3.1	1.9, 4.7	3.70	0.056
Meningitis	18.2	15.3, 21.4	34.0	30.4, 37.8	40.1	<0.001
Dysentery	6.3	4.6, 8.4	0.2	0.0, 0.8	37.3	<0.001
Diarrhea	19.5	16.5, 22.8	2.3	1.3, 3.7	95.2	<0.001
Pertussis	0.3	0.1, 1.1	8.4	6.4, 10.8	48.4	<0.001
Pneumonia	11.8	9.4, 14.5	16.1	13.4, 19.2	4.9	0.027
Malaria	29.0	25.6, 32.7	18.9	15.9, 22.1	17.6	<0.001
Malaria + Pneumonia	40.8	37.0, 44.7	35.0	31.3, 38.8	4.4	0.035
Hemorrhagic fever	0.8	0.3, 1.8	0.2	0.0, 0.8	1.5	0.220
Other infections	2.1	1.2, 3.5	11.5	9.1, 14.1	43.0	<0.001
Other	0.0	0.0, 0.5	0.0	0.0, 0.5	0.0	1.000
Unspecified	4.5	3.1, 6.4	2.4	1.4, 3.9	4.1	0.044

EAVA – expert algorithm verbal autopsy, PCVA – physician–certified verbal autopsy

*Mid–p 95% confidence limits.

**Table 8** displays the level of agreement between the combined ‘algorithmic cause’ and overall ‘physician–cause’ of death for each diagnosis. There was excellent agreement, with a kappa greater than 0.80, for dysentery, and good agreement, with a kappa greater than 0.60, for diarrhea, both of whose levels as the main cause diverged substantially; as well as good agreement for three additional causes. Four causes had a kappa greater than 0.40, indicating moderate agreement, including pneumonia and meningitis, whose EAVA and PCVA levels as the main cause differed so greatly. Measles and malaria had fair agreement, with a kappa greater than 0.20. Only two causes (Pertussis and other infections) had less than chance agreement, with the lower 95% confidence limit below 0. The kappa measurements at the bottom of **Table 8** show fair to good agreement between some related EAVA and PCVA diagnoses that provide insight into some of the differences in the two methods’ selections of primary and underlying causes of death.

**Table 8:** Agreement of all combined expert algorithm VA primary and possible co–morbid diagnoses with all combined physician–certified VA direct, underlying and contributing causes of 620 child deaths, Niger, 2007–2010

Diagnosis EAVA / PCVA	EAVA	PCVA	Agree +	Agree –	Kappa*	95%CL†
Injury / Injury	6	10	4	609	0.56	0.25, 0.86
AIDS / AIDS	17	10	8	600	0.55	0.32, 0.77
Malnutrition / Malnutrition	145	130	107	451	0.71	0.64, 0.78
Measles / Measles	9	31	6	586	0.28	0.09, 0.46
Meningitis / Meningitis	129	234	128	385	0.59	0.53, 0.66
Dysentery / Dysentery	76	58	58	544	0.85	0.78, 0.91
Diarrhea / Diarrhea	220	189	151	363	0.61	0.55, 0.68
Pertussis / Pertussis	11	64	4	550	0.09	–0.01, 0.19
Pneumonia / Pneumonia	242	143	123	358	0.49	0.42, 0.56
Malaria / Malaria	223	117	87	367	0.35	0.28, 0.43
Hemorrhagic fever / Hem. fever	29	18	15	588	0.62	0.45, 0.78
Other infections / Other infections	122	71	4	431	–0.12	–0.17,–0.07
Unspecified / Unspecified	28	17	15	590	0.66	0.49, 0.82
Possible malaria / Malaria	91	117	79	490	0.71	0.64, 0.78
Other infections / Meningitis	122	234	99	362	0.40	0.32, 0.47

EAVA – expert algorithm verbal autopsy, PCVA – physician–certified verbal autopsy;

*Kappa agreement: Less than chance 10, Slight ≤0.20, Fair ≥0.21, Moderate ≥0.41, Good ≥0.61, Excellent ≥0.81.

†Mid–p 95% confidence limits.

**Figure 3:**
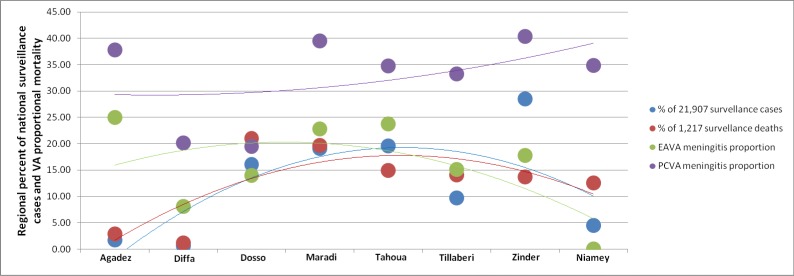
Meningitis surveillance cases and deaths, and verbal autopsy expert algorithm hierarchical primary and physician certified underlying meningitis deaths, Niger, 2007–2010.

**Geographic distribution of surveillance and VA meningitis.** **Figure 3** displays the percent of all meningitis cases and deaths identified by the Niger public health surveillance system from 2007 to 2010 in each of the country’s eight regions. The public health laboratory received a CSF sample for 41 percent of the 18 873 cases in 2007 to 2009, of which 45 percent grew out a positive bacterial culture. The pattern was similar by region, suggesting that the surveillance data provide an accurate measure of the distribution of meningitis in Niger during the period of the VA study. **Figure 3** also shows the EAVA– and PCVA–determined meningitis–specific proportional mortality of child deaths in each region identified by the NNMS from 2007 to 2010.

The surveillance data are for all ages, while the VA data are only for children. Also, the surveillance data come from a passive system, while the VA data are for deaths actively identified by a representative household survey. Therefore, it should not be expected to find perfect confluence between the two data sources. Nevertheless, **Figure 3** demonstrates a positive association between the surveillance data and the EAVA findings. The only strongly aberrant data point is for Agadez, where the NNMS detected only eight all–cause child deaths, two of which were EAVA–assessed as due to meningitis. **Figure 3** does not show a relationship between the surveillance data and PCVA meningitis diagnoses.

## DISCUSSION

This verbal autopsy study was conducted as part of CHERG’s effort to directly measure the causes of neonatal and child deaths in several high priority sub Saharan African countries to improve national, regional and global estimates that currently are based mainly on statistical models. This was the first national level CHERG VASA study to be undertaken. The deaths were identified by complete birth histories administered to all women aged 15 to 49 years participating in a national household survey; and, as such, constitute representative samples of neonatal and child deaths during the study’s reference period of 2007 to 2010 from which direct measurements of the causes of death can be made without utilizing a modeling approach.

We conducted verbal autopsy interviews of the deaths and used two analytic methods to determine the causes of death, including VA expert algorithms arranged in a hierarchy to select the primary cause while simultaneously identifying possible co–morbid diagnoses, and physician certification to determine the underlying, direct and contributing causes of death.

For neonatal deaths, the two analytic methods provided broadly similar pictures of the cause proportions of mortality, with severe neonatal infections predominating over other causes, followed by birth asphyxia. Other causes played a less important role according to both methods, although EAVA distinguished more of the less common causes such as diarrhea, tetanus and malformations, while PCVA gave correspondingly greater prominence to severe infections.

The two methods provided more varied pictures of the causes of child deaths. The considerable difference found in the malaria proportions, together with the more similar proportion for malaria combined with pneumonia, could be due to the overlap in the clinical presentations of these conditions [30], with varying interpretation of the findings by the two VA methods. Diarrhea and dysentery were more common by the EAVA method, while meningitis, other infections and Pertussis were more prominent according to PCVA.

The order in which EAVA diagnoses are arranged in a hierarchy can strongly affect the distribution of the causes of death [31]. The hierarchy for the current study was arranged with two principles in mind—first, for early neonatal deaths and for all others, respectively, to identify the main disease or condition of the neonate and the underlying cause of death, which is the cause reported in international mortality statistics; and when this may not be possible, such as when choosing between co–morbid pneumonia and meningitis, to select the cause that typically results in the more severe illness and so is more likely to kill or has the more specific syndrome and so more certain diagnosis. Thus, the aim was to duplicate as closely as possible the causes of death that would be reported according to ICD–10 rules.

Expert algorithms arranged in a hierarchy have been used in several recent national and sub–national verbal autopsy studies of the major causes of neonatal and child deaths [2,26,32,33]. Although the hierarchies for the current study were developed independently of those used by prior studies, there are several similarities between them. This underscores the attention to similar principles likely paid in developing the past and current hierarchies.

For neonatal deaths, one major difference is that the earliest hierarchy, from the 2004 Bangladesh Demographic and Health Survey (DHS) [2], like the current study and in accordance with ICD–10 rules, placed serious infections above preterm delivery, whereas the other studies placed preterm above serious infections or sepsis [26,32], with the study from India even placing preterm above birth asphyxia [33]. Absent other factors, then, all but the Bangladesh DHS study would be expected to find a higher proportion of preterm delivery as the main cause of death than the current study. Unlike the earlier studies, the current study also included meningitis, neonatal jaundice, hemorrhagic disease of the newborn and sudden unexplained death, as we sought to examine whether VA can reliably make these diagnoses when examining all major causes of neonatal death.

The past and current studies’ hierarchies for child deaths also are broadly similar, though the current study again attempted to diagnose conditions not previously examined, including AIDS, underlying malnutrition, dysentery, Pertussis, malaria and hemorrhagic fever. Lastly, both the neonatal and child hierarchies placed diarrhea ahead of pneumonia to keep with WHO interpretation of the ICD coding rule for classifying these conditions when co–morbid.

The physician’s goal when completing a death certificate is to select the main or underlying cause of death, as well as to identify the direct and contributing causes. In part 3 of the certificate the physician denotes the timing of the onset of each cause prior to death, with each antecedent cause required to precede the diagnoses above it, and the underlying cause, on the lowest line, required to be the most distal in onset. This offers a theoretical advantage to the PCVA method; however, few of the VA questions provide information on the timing of the illness sign or symptom’s onset. Therefore, in cases with comorbidity the physician’s judgment plays a large role in the ordering of the causes on the certificate and hence the designation of the underlying cause of death.

Despite all the potential sources of variability in the EAVA and PCVA diagnoses, as mentioned above the two methods identified broadly similar cause distributions for the neonatal deaths and several similarities for the child deaths. This is likely due, first, to the requirement that the physician utilize predetermined minimum diagnostic criteria, thereby imposing some measure of objectivity and standardization on the physician’s diagnoses, similar to this aspect of the expert algorithms, and second, that the EAVA hierarchies were arranged as much as possible according to the same ICD rules that the physician was to follow in filling the death certificates. PCVA analysis has not always included required minimum diagnostic criteria nor completion of a death certificate, which might help explain the large differences found by some other studies between PCVA diagnoses and those reached by other methods [10,34].

For neonates, PCVA diagnosed more sepsis, pneumonia and meningitis than did EAVA. The placement of meningitis and pneumonia above sepsis in the hierarchy decreased the number of primary sepsis cases diagnosed by EAVA, but this does not explain the lower number of EAVA meningitis and pneumonia cases. PCVA meningitis required, at the minimum, the presence of bulging fontanelle or convulsions, while in addition the algorithm required lethargy or unconsciousness. This would tend to lower the number of EAVA meningitis cases. Regarding the greater proportion of deaths classified by EAVA as due to diarrhea, the kappa for all diarrhea diagnoses was 0.71 (95% CL: 0.57, 0.85), indicating good overall agreement between the two methods and suggesting that much of the difference was due to the methods’ varied selection of the primary cause. This appears to be true for several other conditions as well, as there was a moderate or good kappa level of agreement between the combined algorithmic and physician diagnoses for nearly half the neonatal causes of death.

Much the same can be said for the differences in the EAVA primary causes and PCVA underlying causes of child deaths. Overall, there was moderate or good agreement between nine of the 13 causes, indicating that many of the differences were due to the selection of the main cause from among all the diagnoses reached by each method. Nevertheless, closer inspection reveals additional reasons for some of the differences. PCVA pneumonia required at least difficult or fast breathing, while the EAVA required minimum durations for these same illness signs; and even EAVA ‘possible pneumonia’ required additional criteria. Hence, a possible explanation for the larger number of PCVA pneumonia cases is that the physician often diagnosed pneumonia based on the minimum criteria alone. On the other hand, EAVA may have over–diagnosed malaria, given that several of these cases were ‘possible malaria’ (fever and no other VA infectious diagnosis). However, this is balanced by the higher kappa level of agreement of PCVA malaria with EAVA possible malaria than with EAVA malaria, which can only be due to many of the physician’s malaria diagnoses having been based solely on the presence of the minimum required criterion of fever.

The high PCVA meningitis proportion seems likely to be in excess, since its moderate kappa level of agreement with EAVA other infections shows that several PCVA meningitis diagnoses were based on the only overlapping required illness sign, convulsions. This was in addition to the many PCVA meningitis diagnoses that agreed with EAVA meningitis, which required stiff neck or bulging fontanelle. This conclusion is supported by the comparison of the geographic distributions of EAVA and PCVA meningitis to that of the meningitis surveillance data, which showed that EAVA meningitis closely paralleled the surveillance findings while the PCVA levels were consistently higher than the surveillance data. This ecologic assessment also served as a plausibility check of the EAVA child meningitis diagnoses and suggests that the EAVA can provide an accurate estimate of the meningitis–specific proportional mortality of children.

The significant positive association between EAVA maternal infection and early onset neonatal infection strengthens the plausibility of the EAVA diagnosis of severe neonatal infection. It is telling that this relationship held for early onset meningitis, sepsis and all severe infections combined, but not for early onset pneumonia, since it is known from validation studies that pneumonia is one of the more difficult neonatal diagnoses to make by verbal autopsy [8,10,35]. We are not aware of any prior verbal autopsy study that similarly examined such internal associations, thereby strengthening the credibility of the EAVA diagnoses. This plausibility analysis was not deemed feasible for the same PCVA diagnoses due to the inherent risk of bias in the physician finding maternal sepsis whenever early onset neonatal sepsis was diagnosed.

This highlights one potential advantage of EAVA over PCVA, which is its total objectivity and absolute consistency in applying its pre–defined diagnostic criteria [36]. This also might help explain why EAVA identified more of the less common neonatal diagnoses than did PCVA, that is, due to its total objectivity EAVA is open to all possible diagnoses for each case. The physician’s perspective also can be seen in the small number of child diarrhea and dysentery deaths that were diagnosed. Though it’s not possible to categorically state from this study which of the diarrhea estimates is more accurate, the PCVA value of 2.5% is far below the most recent CHERG modeled estimates of the 1 to 59–month diarrhea mortality proportion of 15.6% in Niger and 14.8% in sub–Saharan Africa [37], while the EAVA value of 25.8% is above these estimates.

An unexpected finding, at least on initial examination, both for EAVA and PCVA, was the low proportion of neonatal deaths caused by preterm delivery, respectively, 2.6% and 2.2%. Even including all preterm comorbidity, the EAVA and PCVA levels were, respectively, 9.1% and 7.1%. This compares to CHERG’s 2013 modeled estimates of 31.3% of neonatal deaths in Niger and 30.5% in sub–Saharan Africa [37].

Possible reasons for the large differences between the current findings and the modeled estimates include: 1) falsely low reporting of short pregnancy duration by the current study subjects (both the EAVA algorithm and PCVA minimum criteria required pregnancy duration of less than 8 months or less than 9 months plus symptoms of respiratory distress syndrome), 2) placement of preterm delivery at the bottom of the EAVA hierarchy, whereas the CHERG practice has been to accept the published causes of VA study input data (some of which may have used hierarchies with preterm placed higher up to select among multiple causes) except when more than one underlying cause was given and then to use a hierarchy with preterm delivery above all causes other than congenital abnormalities and neonatal tetanus [38], and 3) acceptance by the CHERG model of less rigorous diagnostic criteria or case definitions, such as ‘prematurity’, without documentation of pregnancy duration or birth weight [38].

The impact of differently arranged hierarchies to select the main cause of death from among multiple possible causes can be illustrated by comparing the current study’s EAVA preterm mortality proportion to the levels in the west African subset of the CHERG model’s VA input data. The model for the causes of neonatal death in high mortality countries with poor quality vital registration data, such as Niger, is based on regression of national level covariates data on the relationship between the same covariates and the cause–specific mortality findings of 112 verbal autopsy studies from throughout the world. Nine of these studies are from west African countries, with the proportion of neonatal deaths caused by preterm delivery ranging from 7.3% to 40.6%, and a mean value of 20.8% [39]. If preterm delivery were placed near the top of the current study’s EAVA hierarchy, then its level (8.8%) would fall on the low end of these other studies’ findings, and so does not appear so “unexpected” as at first glance. Thatte el al’s reanalysis of data on the causes of neonatal death in India illustrates the same point. The original study, with preterm delivery near the top of the neonatal causes of death hierarchy, found that 26.9% of neonatal deaths were caused by preterm delivery [33], while moving preterm to the bottom of the hierarchy decreased its mortality proportion to 9% [31].

Another example can be given of the CHERG model estimates for preterm delivery as a cause of neonatal death appearing to be excessively high when compared to direct measures, but in this case not from a difference in the hierarchies used. The CHERG model estimate for the preterm neonatal mortality proportion in Bangladesh from 2000 to 2013 ranged from 26.1% to 31.2% [37]. The 112 VA studies providing input data to the model included thirteen studies from Bangladesh, with preterm mortality proportion ranging from 0% to 57.2%, median of 18.6% and even the third quartile value, at 24.1%, below the modeled estimate [39]. This example illustrates a question that must be answered through further inquiry and direct, large–scale measures of the causes of neonatal and child mortality in countries with incomplete vital registration data.

Limitations of our study included, first, the well–documented limitations in the validity of all verbal autopsy diagnoses. Much work is ongoing to determine which VA analysis method or combination of methods provides the most valid and reliable diagnoses, but in the meantime VA has proven to be the best source of population–based cause of death data in settings with incomplete vital registration. A second limitation of our study was the long recall period of up to five years that was due to our identifying deaths and conducting VA interviews in the context of a retrospective survey and the need to include a sufficient sample size of deaths. Adequate recall of illness signs and symptoms to determine the population distribution of the causes of children’s deaths for up to 18 months after death has been documented [40]; still, the effects of diminishing memory with time may have compromised the validity of our findings. This same potential limitation will invariably be present with large–scale VA studies taking a retrospective survey approach, so research to examine the actual effect of such a lengthy recall period is warranted.

In summary, we conducted a national level verbal autopsy study to provide direct estimates of the causes of neonatal and child deaths in Niger. These data can be used on their own to supplement modeled estimates of cause–specific mortality; as well as incorporated into modeling exercises to improve modeled mortality estimates. Further studies are warranted to examine whether direct measures from well–conducted nationally–representative verbal autopsy assessments or modeled estimates of the causes of death are to be preferred. This was not a validation study and so could not definitively determine which analytic method, EAVA or PCVA, provided the most accurate estimates of the cause proportions of mortality. However, the plausibility of the diagnosis of early onset neonatal infection, established through its close association with maternal infection, and the ecological plausibility check of the diagnosis of child meningitis, suggest that at least for these two diagnoses the EAVA method is to be preferred. Validation studies are needed to fully assess this method’s validity and to compare the method to the newer statistical approaches to VA analysis, a comparison which has yet to be performed.
