# Supplementary material for: Direct estimates of national neonatal and child cause–specific mortality proportions in Niger by expert algorithm and physician–coded analysis of verbal autopsy interviews
Source: J Glob Health. 2015 Apr 25;5(1):010415. doi: 10.7189/jogh.05.010415 (PMC4416334; doi:10.7189/jogh.05.010415)
Supplement: Online Supplementary Document [file jogh-05-010415-s001.pdf]

Online Supplementary Document

Kalter et al. Direct estimates of national neonatal and child cause–specific mortality proportions in Niger by expert algorithm and physician–coded analysis of verbal autopsy interviews

J Glob Health 2015;5:010415

Appendix 1: Verbal autopsy expert algorithms for neonatal and child causes of death and for maternal infection

Neonatal causes of death

- Neonatal tetanus
  - (Age 3–27 days at death AND convulsions or spasms)
    AND EITHER
    ((Able to suckle normally during the first day of life and stopped being able to suckle) OR (cried within 5 minutes after birth and stopped being able to cry))

- Congenital malformation
  - Gross malformation present at birth

- Birth asphyxia
  - Neonatal respiratory depression: (Did not cry within 5 minutes after birth OR did not breathe immediately after birth)
    AND
    Neonatal encephalopathy: (Not able to suckle normally in the first day of life OR convulsions/spasms OR lethargy) OR 0 days old at death

- Birth injury
  - Bruises or signs of injury on the body at birth

- Preterm delivery with respiratory distress syndrome (combined with preterm for final cause distribution)
  - Pregnancy duration less than 9 months
    AND
    (Fast breathing starting on day 0 AND no fever AND no cold to touch)

- Meningitis
  - Fever AND (bulging fontanelle OR convulsions) AND (lethargic OR unresponsive/unconscious)

- Diarrhea

    - More frequent loose or liquid stools than usual AND more than 4 stools on the day the diarrhea was most frequent

- Pneumonia
    - (Fast breathing lasting 1 day or more OR difficult breathing lasting 1 day or more and lasting until death)
      AND
      2 or more of the following 3 signs: (chest indrawing, grunting, never cried OR stopped crying)

- Possible diarrhea (combined with diarrhea for final cause distribution)
    - More frequent loose or liquid stools than usual AND VA sepsis (see below) AND No VA diarrhea

- Possible pneumonia (combined with pneumonia for final cause distribution
    - Difficult breathing AND VA sepsis AND No VA pneumonia

- Sepsis
    - Fever OR cold to touch
      OR
      2 or more of the following 7 signs: (fever OR cold to touch, did not suckle normally on the first day of life OR stopped suckling, convulsions, vomited everything, stopped crying, lethargic OR unconscious, chest indrawing OR grunting)

- Neonatal jaundice
    - Yellow skin or yellow eyes AND (stopped being able to suckle normally OR lethargic OR unresponsive/unconscious) AND No fever or hypothermia

- Neonatal hemorrhagic syndrome
    - Bleeding from anywhere AND No fever or cold to touch

- Sudden unexplained death
    - Died suddenly without appearing ill AND No illness signs or symptoms

- Preterm delivery
    - Pregnancy duration less than 8 months

- Unspecified (all others)
    - All VA diagnoses are negative

Child causes of death

- Injury
    - Suffered from motor vehicle accident, fall, drowning, poisoning, venomous bite or sting, burn, violence or other injury
      AND
      (Died 1 day or less after the injury AND the illness lasted 1 day or less) OR (Injury and No other VA diagnosis (except malnutrition allowed)) OR (Injury that was the first illness sign/symptom AND had VA other infection or fever))

- AIDS
    - (Swelling in the armpits OR a whitish rash inside the mouth/on the tongue)
      AND
      3 or more of the following 6 signs: (limbs became very thin, protruding belly, more frequent loose/liquid stools than usual for more than 30 days, fever or a skin rash for more than 30 days, fast breathing, chest indrawing)

- Malnutrition (underlying)
    - Limbs became very thin during the fatal illness OR had swollen legs or feet during the illness
      AND
      One of these was the first symptom of the illness

- Measles
    - Child's age greater than or equal to 120 days AND rash for 3 or more days AND fever for 3 or more days AND the rash started on the face

- Meningitis
    - Fever AND (stiff neck OR bulging fontanelle)

- Dysentery
    - More frequent loose or liquid stools than usual AND more than 4 stools on the day with the most stools AND blood in the stools
      OR
      More frequent loose or liquid stools than usual for more than 14 days AND blood in the stools

- Diarrhea
    - More frequent loose or liquid stools than usual AND more than 4 stools on the day with the most stools AND No blood in the stools
      OR
      More frequent loose or liquid stools than usual for more than 14 days AND No blood in stools

- Pertussis

- Cough more than 14 days <u>AND</u> (severe cough <u>OR</u> vomited after coughing <u>OR</u> stridor)

- Pneumonia
  - (Cough more than 2 days <u>OR</u> difficult breathing more than 2 days)
    <u>AND</u>
    (Fast breathing more than 2 days <u>OR</u> chest indrawing <u>OR</u> grunting)

- Malaria
  - Fever that continued till death <u>AND</u> was on and off in character <u>AND</u> No stiff neck <u>AND</u> No bulging fontanelle <u>AND</u> (pallor <u>OR</u> difficult breathing <u>OR</u> convulsions <u>OR</u> unconscious till death)
    <u>OR</u>
    Fever that continued till death <u>AND</u> was severe fever <u>AND</u> No stiff neck <u>AND</u> No bulging fontanelle <u>AND</u> (pallor <u>OR</u> convulsions <u>OR</u> unconscious till death)

- Possible dysentery (combined with dysentery for final cause distribution)
  - More frequent loose or liquid stools than usual <u>AND</u> (fever OR convulsions OR unconscious up till death) <u>AND</u> blood in the stools <u>AND</u> No VA dysentery

- Possible diarrhea (combined with diarrhea for final cause distribution)
  - More frequent loose or liquid stools than usual <u>AND</u> (fever OR convulsions OR unconscious up till death) <u>AND</u> No blood in the stools <u>AND</u> No VA diarrhea

- Possible pneumonia or ARI (combined with pneumonia for final cause distribution)
  - (Cough or difficult breathing) <u>OR</u> (Fast breathing <u>AND</u> (chest indrawing OR stridor OR grunting OR wheezing))
    <u>AND</u>
    (Severe cough <u>OR</u> post–tussive vomiting <u>OR</u> fast breathing <u>OR</u> chest indrawing <u>OR</u> grunting <u>OR</u> stridor <u>OR</u> wheezing <u>OR</u> fever <u>OR</u> convulsions <u>OR</u> unconscious up till death) <u>AND</u> No VA Pertussis <u>AND</u> No VA pneumonia

- Hemorrhagic fever
  - Fever <u>AND</u> (bled from anywhere <u>OR</u> had areas of the skin that turned black)

- Other infection
  - Fever <u>AND</u> (rash on trunk, abdomen or everywhere <u>OR</u> convulsions <u>OR</u> unconscious up till death)

- Possible malaria (combined with malaria for final cause distribution)
  - Fever <u>AND</u> No other VA infectious causes of death

- Malnutrition (combined with underlying malnutrition for final cause distribution)

    - Limbs became very thin during the fatal illness <u>OR</u> had swollen legs or feet during the illness

- Unspecified (all others)
    - All VA diagnoses are negative

<u>Maternal complication</u>

- Maternal infection during pregnancy
    - Fever <u>AND</u> (severe abdominal pain <u>OR</u> smelly vaginal discharge)

- Maternal infecton during labor and delivery
    - Fever <u>AND</u> (severe abdominal pain <u>OR</u> smelly vaginal discharge <u>OR</u> foul smelling liquor)

Appendix 2: Verbal autopsy hierarchies

<u>Neonatal causes of death</u>

Neonatal tetanus
Congenital malformation
Birth asphyxia or Birth injury
Preterm delivery with respiratory distress syndrome (combined with preterm delivery for final cause distribution)
Meningitis
Diarrhea
Pneumonia
Possible diarrhea (combined with diarrhea for final cause distribution)
Possible pneumonia (combined with pneumonia for final cause distribution)
Sepsis
Neonatal jaundice
Neonatal hemorrhagic syndrome
Sudden unexplained death
Preterm delivery
Unspecified (all others)

<u>Child causes of death</u>

Injury (from motor vehicle accident, fall, drowning, poisoning, venomous bite or sting, burn, violence or other)
AIDS
Malnutrition (underlying)
Measles

Meningitis
Dysentery
Diarrhea
Pertussis
Pneumonia
Malaria
Possible dysentery (combined with dysentery for final cause distribution)
Possible diarrhea (combined with diarrhea for final cause distribution)
Possible pneumonia (combined with pneumonia for final cause distribution)
Hemorrhagic fever
Other infections
Possible malaria (combined with malaria for final cause distribution)
Malnutrition (combined with underlying malnutrition for final cause distribution)
Unspecified (all others)

Appendix 3: Physician minimal diagnostic criteria

<u>Neonatal causes of death</u>

- Neonatal tetanus
    - Stopped suckling normally more than 2 days after birth <u>AND</u> Spasms or convulsions

- Congenital malformation
    - Physical abnormality at the time of delivery

- Birth asphyxia
    - Did not breath immediately after birth <u>OR</u> Did not cry immediately after birth

- Birth trauma
    - Bruises or signs of injury at birth

- Preterm delivery with respiratory distress syndrome
    - (Pregnancy duration less than 9 months <u>OR</u> Pregnancy ended early)
<u>AND</u>
(Fast breathing <u>AND</u> No fever <u>AND</u> No cold to touch)

- Meningitis (part of "Serious Infection")
    - Bulging fontanelle <u>OR</u> Spasms or convulsions

- Diarrhea
    - More frequent loose or liquid stools than usual

- Pneumonia (part of “Serious Infection”)
  - Difficult breathing OR Fast breathing

- Sepsis (part of “Serious Infection”)
  - (Fever OR Cold to touch) AND (No diagnosis of pneumonia or meningitis)

- Preterm delivery
  - Pregnancy duration less than 8 months OR Pregnancy ended early

- Unspecified (Unknown)
  - Does not meet any of the above criteria AND No other specified diagnosis

Child causes of death

- Injury (Venomous, Drowning, Fall, Fire, Poisoning, Road traffic, Violent, Unspecified)
  - Suffered an injury or accident

- AIDS
  - Whitish rash of tongue or mouth OR Swelling in the armpits

- Malnutrition (severe)
  - Limbs became very thin OR Swollen legs or feet OR Protruding belly

- Measles
  - Fever AND Rash

- Meningitis
  - Stiff neck OR Bulging fontanelle OR Generalized convulsions or fits

- Dysentery
  - More frequent loose or liquid stools than usual AND Visible blood in the stools

- Diarrhea
  - More frequent loose or liquid stools than usual

- Pertussis
  - Severe cough

- Pneumonia
  - Difficult breathing OR Fast breathing

- Malaria

    - Fever

- Hemorrhagic fever
    - Bleeding from anywhere OR Skin turned black

- Other childhood infectious disease (not specified above)
    - Fever AND Infectious diagnosis not specified above

- Unspecified (Unknown)
    - Does not meet any of the above criteria AND No other specified diagnosis
